# Utility of hybrid SPECT-CT in the detection of unsuspected single lytic vertebral metastases in renal cell carcinoma

**DOI:** 10.4103/0972-3919.63600

**Published:** 2010

**Authors:** CNB Harisankar, Bhagwant Rai Mittal, Anish Bhattacharya, Baljinder Singh

**Affiliations:** Department of Nuclear Medicine, Postgraduate Institute of Medical Education and Research, Chandigarh, India

**Keywords:** Bone scan, metastasis, renal cell carcinoma, SPECT-CT

## Abstract

Authors describe the incremental value of hybrid SPECT-CT in upstaging disease and changing the management strategy of a case of renal cell carcinoma

## INTRODUCTION

Bone scintigraphy is less sensitive for detection of osteolytic lesions. We describe the utility of hybrid modality SPECT-CT in demonstrating the osteolytic lesion on morphological (CT) component of SPECT-CT associated with focal subtle tracer uptake.[1‐3]

## CASE REPORT

**Figure 1:**
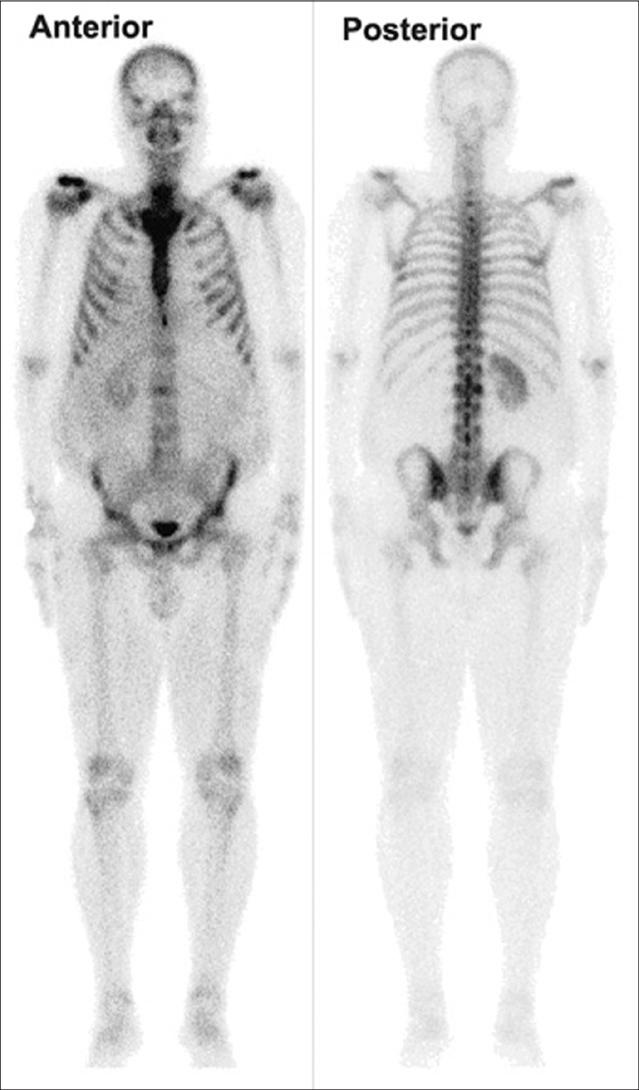
Planar bone scintigraphy performed after I.V. injection of technetium labeled MDP showing very mild increase in the L 2 vertebra prompted a hybrid SPECT-CT imaging of the lumbar spine. Post Left nephrectomy status is also noticed

**Figure 2:**
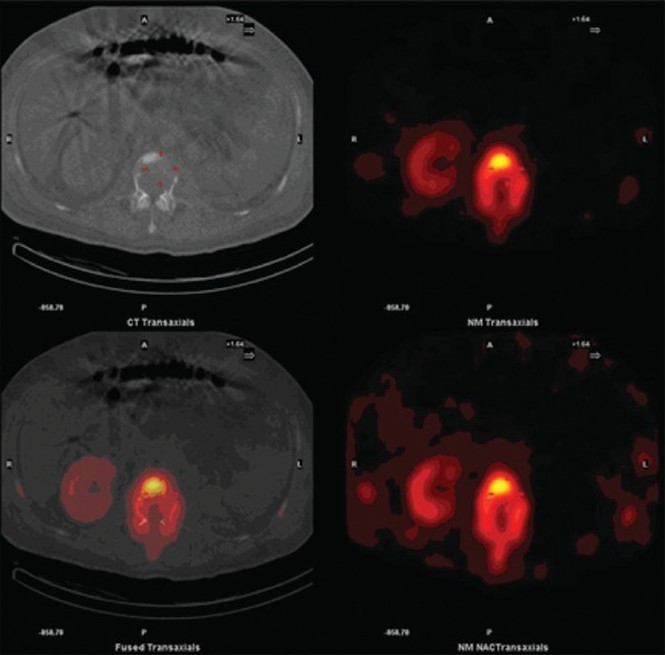
Hybrid SPECT-CT images revealing cortical destruction of the L2 vertebra. The photopenic region in SPECT images corresponding to the site of destruction

We report the case of a 47-year-old male patient who presented with abdominal pain, lower urinary symptoms and hematuria for three months. He was diagnosed as a case of renal cell carcinoma and subjected to radical nephrectomy. The patient also underwent a whole body bone scan for a metastatic workup. A planar whole body bone scan along with a hybrid single photon emission computed tomography (SPECT) / Computed Tomography (CT) imaging was performed. The planar imaging showed an area of mildly increased tracer uptake in the second lumbar vertebra [Figure 1]. Subsequent SPECT / CT imaging revealed a lytic lesion in the L2 vertebra, with increased uptake in the rest of the vertebral body; the findings more likely to be with metastatic involvement [Figure 2]. Thus, SPECT / CT upstaged the disease and changed the management strategy. In conclusion the present case report emphasizes the importance of a hybrid SPECT / CT imaging for suspicious bone lesions on planar images.
